# Mucosal immunity in cancer metastasis: roles, mechanisms, and therapeutic implications

**DOI:** 10.3389/fimmu.2026.1809558

**Published:** 2026-03-27

**Authors:** Yaru Miao, Dongdong Cui, Yanghui Bi, Ruiping Zhang

**Affiliations:** 1 Institute of Medical Technology, Shanxi Medical University, Taiyuan, China; 2 Academy of Medical Sciences, Shanxi Medical University, Taiyuan, China; 3 Shanxi Bethune Hospital Gene Sequencing Center, Shanxi Academy of Medical Sciences, Taiyuan, China; 4 The Radiology Department of Shanxi Provincial People’s Hospital Affiliated to Shanxi Medical University, Taiyuan, China

**Keywords:** immune tolerance, microbiota, mucosal immunity, pre-metastatic niche, tissue-resident immunity, tumor metastasis

## Abstract

Previous studies have shown that the mucosal immune system plays a crucial role in regulating immune tolerance, maintaining the integrity of the mucosal barrier, and facilitating immune communication between organs. However, its role in tumor metastasis has not been fully investigated. This review integrates recent studies on mucosal immunity and tumor metastasis, systematically discussing the process by which mucosal immune dysregulation promotes tumor metastasis. We elucidate how the mucosal imprinting program formed within the common mucosal immune system affects metastasis through regulating immune tolerance, tissue-specific homing, and the circulating adaptability of metastatic tumor cells. During this process, the microbiota within the tumor also plays an important synergistic role. Through major mucosal axes such as the gut-lung axis and the gut-liver axis, damaged mucosal immunity remodels the composition and metabolic state of tissue-resident immune cells and remotely regulates the pre-metastatic microenvironment. At the distal mucosal sites, immune populations such as alveolar macrophages and tissue-resident memory T cells inhibit tumor metastasis growth by forcing tumor dormancy, maintaining mucosal immune balance. However, when the body experiences a chronic infection and the mucosal immune system is disrupted, dormant tumor cells can be reactivated for metastasis. This review describes the roles of mucosal immunity at different stages of tumor metastasis, providing a reference for understanding the role of mucosal immunity in tumor metastasis and revealing the key mechanism pathways and therapeutic strategies for preventing metastasis recurrence.

## 1 Introduction

In recent years, significant progress has been made in the treatment of primary tumors. However, tumor metastasis remains the fundamental cause of death for most cancer patients, accounting for over 90% of global cancer deaths. The “seed and soil” hypothesis proposed by Stephen Paget in 1889 laid the theoretical foundation for the study of the tumor microenvironment in the occurrence and development of tumors (1). With the development of high-throughput sequencing, spatial multi-omics and microbiomics, the concept of “soil” has gradually evolved from a passive description of the micro-environment to a dynamic ecosystem. This system is jointly shaped by the microbial community, extracellular matrix and immune regulation (2, 3). Within this evolving theoretical framework, mucosal tissues hold a unique and crucial position. Mucosal immunity refers to the specialized immune system at barrier surfaces, primarily the gastrointestinal and respiratory tracts, which maintains a delicate balance between defending against pathogens and tolerating commensal microbes. This review primarily focuses on cancers originating from these mucosal epithelia, including colorectal cancer (CRC), non-small cell lung cancer (NSCLC), gastric cancer, nasopharyngeal cancer (NPC), and salivary gland tumors, as well as other systemic malignancies such as breast cancer, melanoma, and pancreatic cancer that frequently exploit mucosal immune axes to metastasize to distant organs like the lungs, liver, and brain (4, 5). Furthermore, primary tumors that originate from mucosal epithelium (such as colorectal cancer, non-small cell lung cancer and nasopharyngeal cancer) often retain the “mucosal imprint” characteristic of their tissue origin (6). Mucosal imprinting is a programming process by which cells acquire tissue-specific homing receptors and metabolic adaptations dictated by their original mucosal microenvironment, a signature that subsequently directs their organ-specific dissemination. This imprint exhibits the following characteristics: the expression of tissue-specific homing receptors and wide adaptability to continuous microbial exposure and strict immune tolerance environments (7, 8). The realization of the above mechanism does not rely solely on the actions of tumor cells, but is jointly achieved by the functional unit composed of tumor cells and their intracellular microbial communities (the “microbial - tumor symbiont”), while the host mucosal immune system provides crucial microenvironmental support for the survival, colonization and adaptive evolution of this symbiont (9–11).

In traditional immunology, the common mucosal immune system (CMIS) is regarded as an important physiological barrier for maintaining mucosal homeostasis. Its core function lies in restricting the systemic spread of pathogens between different mucosal organs (12–14). However, during the process of tumor metastasis, this system may be exploited by tumor cells and become a medium that promotes tumor metastasis and spread (15, 16). The mucosal immune network that regulates immune defense in various mucosal-related organs can be reprogrammed by tumor cells to facilitate their migration and distant colonization (17). At the mechanism level, mucosal immune imprinting provides directional homing signals and microenvironmental permission for disseminated tumor cells (DTCs). Under physiological conditions, lymphocytes achieve directional homing to the intestinal mucosa through integrin α4β7 and chemokine receptor CCR9 and other molecules (18–20). Circulating tumor cells (CTCs) exhibit abnormal expression of the same pathways, thereby also being directed towards mucosal-related organs. Additionally, mucosal tissues are typically rich in regulatory T cells (Tregs) and immunosuppressive cytokines like interleukin-10 (IL-10) that have immunosuppressive functions. Their homeostasis depends on the regulation of the symbiotic microbial community and plays a crucial role in suppressing chronic inflammation. However, this characteristic provides an immunological exemption microenvironment for metastatic tumor cells and their symbiotic microorganisms to some extent (21–23).

Current scientific consensus defines metastasis as a complex, multi-step, and microenvironment-dependent process. Within this paradigm, growing evidence indicates that the tumor microbiome functions as a non-cell-autonomous regulator and integral component of this dynamic ecosystem. Intratumoral microbiota contribute to metastatic progression by driving immunosuppression, promoting epithelial-mesenchymal transition, and enhancing circulating tumor cell fitness, thereby acting as critical co-factors in seeding distant metastases (24, 25). Studies have shown that it is not only tumor cells that are involved in the metastasis process; the intratumoral microorganisms in the primary lesion also act as functional components accompanying the tumor metastasis. During the process of tumor metastasis, microbial components enhance the ability of barrier penetration and reshape the cytoskeletal structure, helping tumor cells acquire mechanical resilience to resist shear stress in the bloodstream, thereby facilitating tumor cell metastasis. In addition to their effects on tumor cells themselves, tumor-associated microorganisms also play a role in shaping the immune-mediated pre-metastatic microenvironment, especially in mucosal-related organs such as the lungs and liver (5, 26). This process is often accompanied by the aggregation of myeloid cells and the formation of neutrophil extracellular traps (NETs). This reticular structure composed of chromatin and granular proteins can effectively capture CTCs, facilitating their retention and subsequent proliferation in distant organs (27, 28).

The above research highlights the significant role of mucosal immunity in the process of tumor metastasis. The mucosal immune system not only provides “soil” for metastatic cells to colonize, but also plays an important regulatory role in the selection of metastatic pathways, the establishment of immune balance, and the maintenance of tumor dormancy. This review will focus on the mucosa-specific immune regulatory mechanisms during tumor metastasis, systematically exploring the following core questions: How do tumor microorganisms interact with the host’s mucosal immune program to initiate and continuously drive the metastasis process? How do the resident immune cells within mucosal organs establish a dynamic immune balance and determine the fate of tumor cells in metastatic lesions? How do acute mucosal infections such as influenza disrupt this balance and subsequently awaken dormant tumor cells with the potential for metastasis? Finally, based on the above mechanisms, we have proposed a new anti-metastasis intervention framework centered on reshaping the mucosal microenvironment: by targeting and disrupting the “microbe-tumor symbiont” relationship, and using mucosal vaccine administration and other methods to reprogram the local immune response, we can systematically inhibit the occurrence of metastasis.

## 2 Mucosal immune disruption as a driver of metastatic dissemination

**Figure 1:**
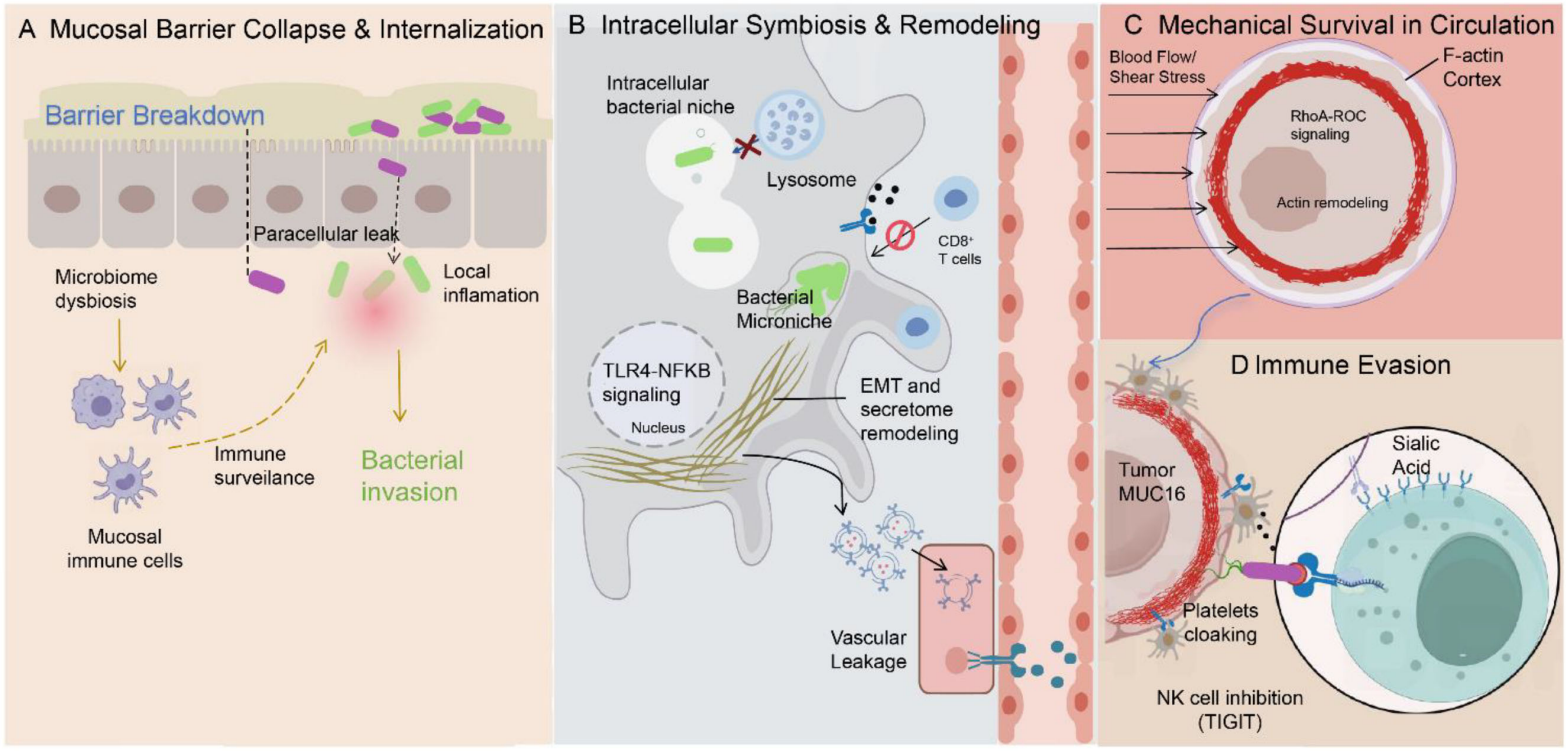
Systematic subversion of mucosal immunity drives metastatic dissemination of mucosa-derived tumors. **(A)** Mucosal barrier collapse enables microbial invasion. Disruption of epithelial integrity and mucosal immune defenses at the tumor–mucosa interface permits bacterial translocation. Microbiota dysbiosis weakens epithelial barrier function and secretory IgA–mediated protection, creating permissive conditions for microbial entry into tumor tissues. **(B)** Intracellular symbiosis reshapes tumor behavior. Following invasion, selected bacteria establish intracellular niches within tumor cells, activating host signaling pathways and promoting epithelial–mesenchymal transition (EMT) and tumor secretome remodeling, thereby facilitating vascular entry and dissemination. **(C)** Biomechanical adaptation supports circulatory survival. Bacteria-containing circulating tumor cells undergo cytoskeletal remodeling that enhances resistance to hemodynamic shear stress, enabling transient persistence during systemic circulation. **(D)** Immune evasion promotes metastatic colonization. The microbe–tumor symbiont exploits mucosal immune tolerance mechanisms to evade innate immune surveillance, including platelet cloaking, immunosuppressive glycan signaling, and inhibition of cytotoxic lymphocytes. Collectively, these processes reflect hijacking of the common mucosal immune system (CMIS) to facilitate metastatic spread.

Research shows that for tumors occurring on mucosal surfaces such as the gastrointestinal tract and respiratory tract, the metastasis and spread in the mucosal area are essentially caused by the disruption of local mucosal immune balance. As shown in **Figure 1**, this process can be broken down into three key components: the resident microbiota, mucosa-specific immune programs, and tumor cells regulated by the primary tissue, all of which play crucial regulatory roles in metastasis. First, the disruption of the mucosal immune barrier leads to microbial invasion, forming a microbiota-tumor symbiosis. Studies in breast cancer and colorectal cancer have shown that specific bacterial species (such as *Fusobacterium nucleatum*) are simultaneously detected in primary mucosal tumors and their distant metastases, indicating their systemic co-migration with tumor cells (29, 30). The formation of this symbiotic unit exacerbates local and systemic immune disorders. The systemic immune tolerance enables this symbiotic unit to survive in the circulation and evade clearance. Further, the distant mucosal immunity is reprogrammed to shape the pre-metastatic niche that allows the metastatic cells to colonize and grow, thereby creating a microenvironment conducive to tumor metastasis.

### 2.1 Mucosal immune barrier failure licenses microbial invasion and symbiotic niche formation

The metastasis of tumors derived from mucosal tissues is not an accidental phenomenon. Its root cause lies in the loss of the physiological barrier function that defends against microorganisms. These barriers include the mucosal immune barrier and the epithelial structural barrier (**Figure 1A**). In the early stage of tumor development, the depletion of short-chain fatty acids (SCFAs) observed in ulcerative colitis, colorectal cancer, and gastric cancer disrupts the epithelial immune homeostasis, leading to the downregulation of junctional proteins, including tight junction components (such as occludin and zonula occludens-1 [ZO-1]) and desmosomal proteins (such as desmoglein-2 [DSG2]), and an increase in cell intercellular spaces (31–33). In addition to disrupting the structure of the mucosal barrier, the depletion of SCFAs can also affect the epithelial defense function through epigenetic regulation. As confirmed by Mann et al. (34), the reduction of short-chain fatty acids alters the histone acetylation pattern of epithelial cells, thereby weakening their ability to respond to microbial invasion (34). Meanwhile, the pathological remodeling of the tumor-associated mucus layer promotes the formation of bacterial biofilms, allowing microorganisms to accumulate in immune-deficient areas and further enhancing their ability to penetrate the basement membrane (35).

The decline of the immune barrier function will further exacerbate this process. Some bacteria can secrete IgA1 protease, which degrades the secretory IgA (SIgA) on the mucosal surface, thereby weakening the defense function of mucosal immunity (36). As the function of the IgA immune barrier weakens, microorganisms undergo selective migration, thereby enabling microorganisms such as Fusobacterium nucleatum to penetrate the basement membrane through lectin-dependent mechanisms and invade the interior of tumor cells. The invasion process of this microorganism is highly selective. Analysis of the microbial community in the core of gastrointestinal tumors indicates that only a few “microbial elites” that can adapt to the low-oxygen and nutrient-poor microenvironment within the tumor can persist and colonize in the metastatic microenvironment (37).

Analysis of large-scale cancer-wide data reveals that the bacterial communities within tumors are mainly dominated by intracellular colonizing bacteria specific to certain cancer types (38). In pancreatic and colorectal cancers, these bacterial communities are mainly distributed in the immune microenvironment of malignant cells and the surrounding stroma (39). It is essential to acknowledge that while intratumoral microbiota are increasingly recognized as a pan-cancer phenomenon, the composition and abundance of these microbial communities are highly cancer-type dependent rather than universal (40, 41). Within the tumor microenvironment, these microbes do not distribute randomly; instead, they colonize specific microniches that are spatially organized to interact with immune and epithelial cells, exhibiting significant intratumoral heterogeneity (42). Furthermore, detecting these populations presents substantial methodological challenges due to their low biomass relative to host tissues. To differentiate true biological symbiosis from environmental contamination, recent high-quality studies emphasize the necessity of rigorous negative controls and multi-modal validation strategies—such as combining 16S rRNA sequencing with spatial transcriptomics or fluorescence *in situ* hybridization (FISH)—to confirm the presence and localization of tumor-associated bacteria (41, 42). When microorganisms enter the interior of tumor cells, they form a protective intracellular microenvironment that helps them resist the attack of the host’s humoral immunity, while also providing specific survival advantages for the tumor cells. Invasive bacteria can actively regulate the autophagy process and anti-apoptotic signaling pathways of host cells, thereby effectively preventing them from being degraded by lysosomes. This mechanism ensures that microorganisms can coexist stably within the cells for a long time, creating favorable conditions for the disruption of the mucosal barrier and the metastasis of tumor cells (43).

### 2.2 Systemic immune tolerance enables circulatory survival of the microbe–tumor symbiont

After successfully establishing intracellular colonization, microorganisms form symbiotic units with tumor cells, promoting tumor metastasis (44). This process involves not only the intrinsic reprogramming of the tumor cells themselves, but also the abnormal extension of the mucosal immune tolerance state to the circulatory system (45) (**Figure 1B**). Studies have shown that bacteria associated with tumors can regulate the expression of host miRNAs and activate inflammatory signaling pathways, thereby initiating the epithelial-mesenchymal transition program (46). This enables epithelial-derived breast cancer cells to acquire stronger migration potential and stem cell-like characteristics. The analysis of spatial transcriptomics further indicates that the tumor regions enriched with bacteria form a highly structured microenvironment, which actively repels cytotoxic CD8^+^ T cells, thereby establishing a local immune-privileged area and helping tumor cells evade immune surveillance by the vascular system (47). Kang and his team proposed the “Trojan Horse” model for breast cancer, which indicates that tumor cells regulated by microorganisms can release extracellular vesicles (EVs) containing bacterial components (48). These EVs carry characteristics of mucosa-related glycosylation modifications such as lipopolysaccharides and α2,6-sialylation (48). In salivary adenoid cystic carcinoma and general metastatic models, these EVs can be taken up by endothelial cells and, by inducing vascular endothelial stress responses or reducing the expression level of vascular endothelial cadherin, they can damage the integrity of the endothelial layer, thereby enhancing vascular permeability and promoting the transendothelial migration process of tumor cells (49, 50).

When the symbiotic units enter the circulatory system, the abnormal activation of the mucosal immune program enables the symbionts to survive (44). Usually, in prostate adenocarcinoma and chronic lymphocytic leukemia, the IgA plasma cells, which serve as the front line of mucosal defense, can be reprogrammed in the tumor microenvironment and instead express molecules such as programmed cell death ligand 1 (PD-L1) and IL-10 with immunosuppressive effects, thereby weakening the anti-tumor T cell response and promoting the spread of tumor cells throughout the body (45, 51). Furthermore, through the “mucosal camouflage layer” composed of secreted IgA, the microbial-tumor symbiosis can mimic benign symbiotic bacteria and utilize the host’s immune tolerance to mucosal antigens to evade recognition by NK cells in the immune system. The tumor-related glycosylation process further enhances this immune tolerance. As highlighted in studies of prostate cancer and other metastatic malignancies, abnormal cell surface sialylation modifications can activate inhibitory Siglec receptors on NK cells and other immune effector cells, thereby actively suppressing the cytotoxic response and extending the mucosal immune tolerance state to the systemic circulation system (52, 53). The above mechanism indicates that the systemic metastasis of tumors originating from the mucosa not only depends on the classic immune escape, but its deeper essence lies in the pathological remodeling of mucosal immune tolerance. This remodeling process was originally intended to maintain the homeostasis balance between the host and the microbial community.

It should be clarified that the degradation of SIgA by bacterial proteases and the formation of the mucosal camouflage layer are not contradictory but rather stage-specific adaptations. In the early invasion stage, pathogens utilize proteases to breach the physiological IgA barrier to establish intracellular colonization. Once the microbe-tumor symbiont is formed and enters the circulation or distal niches, it hijacks host-derived, tumor-reprogrammed IgA—which lacks protective function but retains immune tolerance signals—to cloak itself and evade NK cell recognition. Thus, IgA transitions from a barrier to be breached into a shield for immune evasion during metastatic progression.

### 2.3 Mucosal immune reprogramming shapes pre-metastatic niche formation across organs

Although abnormal mucosal immune tolerance enables tumor cells to survive temporarily in the circulation, the success of tumor metastasis depends on whether the distant target organs possess an immune microenvironment that allows their colonization. For tumors originating from the mucosa, the formation of the pre-metastatic microenvironment is the result of systemic mucosal immune reprogramming (14, 54) (**Figure 1C**). Through the CMIS, immune disturbances originating from a certain mucosal site can spread to organs that are anatomically distant from each other, thereby making the metastasis process tend to occur in specific locations (55).

The core mechanism of this process lies in the imbalance of the basic immunosuppressive state of mucosal organs. The primary disorder of the mucosal microbiota can lead to the depletion of immune regulatory metabolites derived from the intestine, and subsequently, through systemic immune regulatory pathways, remotely alter the functional state of immune cells in the lungs (56). This weakening of the continuous immunosuppressive signal reduces the activation threshold of the innate immune response, thereby creating an inflammatory microenvironment conducive to the retention and survival of tumor cells. Clinical data indicate that mucosal malignant tumors often co-occur with mucosa-associated lymphoid tissue (MALT) lymphoma. This comorbidity phenomenon, observed in papillary thyroid cancer and colonic adenocarcinoma, confirms the systemic association of immune states in different mucosal sites, further demonstrating that chronic mucosal immune disorders promote the formation of a systemic microenvironment suitable for tumor metastasis (57, 58). Furthermore, the microbial-tumor symbiosis can achieve immune evasion through the immune checkpoint pathways, as demonstrated in colorectal cancer. For instance, the outer membrane protein Fap2 of non-fermentative rods is a high-affinity ligand for the inhibitory receptor TIGIT, and TIGIT is widely expressed on the surface of natural killer cells and tumor-infiltrating lymphocytes. The expression of Fap2 can activate the TIGIT-ITIM signaling axis, inhibit the cytotoxic effects of immune cells, and thereby protect metastatic tumor cells from immune-mediated clearance (59, 60).

The aforementioned immunosuppressive microenvironment can further recruit and polarize innate immune cells, facilitating the immune escape of tumor cells. Microbial-derived signals, such as those from the intratumoral microbiota, can trigger the formation of neutrophil extracellular traps (NETs). For instance, in colorectal cancer, components of Fusobacterium nucleatum have been shown to induce NETosis, which facilitates the capture of circulating tumor cells (CTCs) and enhances the local immunosuppressive state (27, 61). The above research results suggest that the formation of the pre-metastatic microenvironment within mucosal organs is regulated by systemic immune reprogramming. This indicates that mucosal immunity plays a crucial role in determining the organ-specific specificity of tumor metastasis.

## 3 Pre-emptive dismantling of mucosal immune surveillance before metastasis

**Figure 2:**
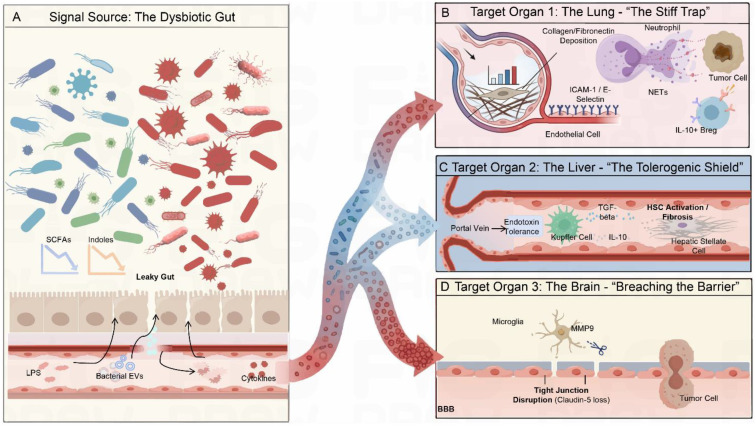
CMIS-mediated pre-emptive dismantling of mucosa-connected immune surveillance before metastasis. **(A)** A dysbiotic gut mucosa is characterized by loss of immunoregulatory microbial metabolites, such as SCFAs and indole derivatives, and expansion of pathobionts. This imbalance disrupts epithelial and vascular barrier integrity (“leaky gut”), permitting systemic dissemination of microbial products, inflammatory cytokines, and bacterial extracellular vesicles. These signals function as upstream drivers of CMIS-mediated immune reprogramming rather than passive consequences of tumor progression. **(B)** Through the gut–lung axis, circulating microbial and inflammatory cues precondition the pulmonary microenvironment before metastatic seeding. Endothelial cells upregulate adhesion molecules (e.g., ICAM-1, VCAM-1, E-selectin), while fibroblast activation increases matrix stiffness. In parallel, neutrophil recruitment and NET formation generate localized zones of impaired immune accessibility, collectively reflecting anticipatory dismantling of pulmonary immune surveillance. **(C)** Along the gut–liver axis, portal delivery of microbial and tumor-associated signals induces endotoxin tolerance in Kupffer cells, promoting secretion of immunosuppressive cytokines such as IL-10 and TGF-β. Concurrent activation of hepatic stellate cells (HSCs) initiates fibrotic remodeling, increasing tissue stiffness and enhancing the mechanical permissiveness of the liver, thereby converting it from an immune filter into a receptive pre-metastatic niche. **(D)** At the distal end of the CMIS network, systemic inflammatory cues activate microglia within the central nervous system. Activated microglia release matrix metalloproteinases (e.g., MMP9) and vascular endothelial growth factor (VEGF), weakening blood–brain barrier (BBB) integrity. This pre-emptive erosion of neuro-immune surveillance lowers the threshold for tumor cell extravasation, reframing brain metastasis as a consequence of prior immune and barrier compromise.

Studies have shown that before the bacterial-carrying CTCs reach the distal organs, the mucosal immune surveillance mechanism in the target tissue has initiated an active reprogramming (62). The remodeling of the distal organs is driven by the microbiota-tumor symbiotic unit at the primary tumor site. This unit achieves immune regulation of the distal organs by releasing tumor derivatives, microbial metabolites, and exosomes containing bacterial components into the systemic circulation (48, 55) (**Figure 2**). Mucosal organs are not independent immune-isolated units but are interconnected through the CMIS. This system, as a distributed signaling network, can coordinate immune responses between different barrier tissues (14). Tumor cells hijack this network, using pathogen-associated molecular components from intracellular bacteria and tumor-derived exosomes, to transfer and convert the local mucosal immune dysregulation state into specific immune tolerance in distant organs (48, 63). Therefore, before the actual metastasis of tumor cells occurs, evidence from breast cancer models and comprehensive reviews covering malignancies such as lung, colorectal, and pancreatic cancers suggest that specific mucosal immune system axes (including the gut-lung axis, gut-liver axis, and gut-brain axis) have become channels that reduce the immune surveillance capacity of the target organs, preparing the ground for tumor metastasis (62, 64). Thus, the formation of the pre-existing microenvironment before the metastasis is not a passive response triggered by tumor cell proliferation, but rather a pre-established immune remodeling process. During this process, the integrity of the mucosal barrier, the effector functions of the immune system, and the normal responses of the matrix gradually weaken. This early dysregulation of mucosal immune control provides a local environment that is conducive to the colonization, survival, and proliferation of the subsequent tumor cells (**Figure 2A**).

### 3.1 The gut–lung axis: pre-emptive metabolic dismantling of pulmonary mucosal immune surveillance

As the main target organ for mucosal tumor metastasis, the lungs play a crucial role in the CMIS (14). Due to long-term regulation by the intestinal-lung axis signals, the mucosal immune system of the lungs has a high sensitivity to the disruption of the distant intestinal microbiota (65, 66) (**Figure 2B**). In malignant tumors that originate from mucous membranes, the microbiota-tumor symbiosis can disrupt the immune surveillance function of the lungs through the gut-lung axis, as demonstrated in studies of chronic obstructive pulmonary disease (COPD), creating favorable conditions for subsequent metastasis colonization (66).

The fundamental mechanisms of gut-lung immune communication, primarily established in models of chronic inflammatory diseases such as COPD, reveal that SCFAs such as butyric acid can promote the activation of regulatory T cells by activating the GPR43 signaling pathway and inhibiting the activity of histone deacetylases. At the same time, it can inhibit the maturation of dendritic cells, thereby enabling the lungs to form a stronger immune tolerance state (66, 67). The dysregulation of the tumor-associated microbiota can lead to a reduction in the aforementioned metabolites, thereby disrupting the immunosuppressive state maintained by these metabolites. Macrophages and fibroblasts in the lung tissue are activated, exerting pro-inflammatory and pro-fibrotic effects (34). At the same time, the number of aryl hydrocarbon receptor (AhR) derived from tryptophan metabolism will also decrease. As observed in studies of allergic airway inflammation and respiratory antiviral immunity, this not only disrupts the integrity of the lung mucosal barrier but also interferes with the immune balance maintained by innate lymphoid cells (ILCs) (68–70). When the intestinal barrier function is impaired, a condition linked to maternal antibiotic exposure and gut-derived histamine signaling, the weakened immune regulatory function mediated by short-chain fatty acids and the loss of mucosal barrier function dependent on AhR will further intensify, thereby leading to systemic immune imbalance (71, 72). The combined effect of these metabolic disorders interferes with the mucosal immune surveillance function of the lungs, making them more vulnerable to bacterial and antigenic invasion.

At the same time as the metabolic immune collapse occurs, there is also a remodeling of the microenvironment of the pulmonary blood vessels. Due to the increased intestinal permeability, microbial products such as lipopolysaccharides can enter the systemic circulation, triggering persistent chronic inflammation, as demonstrated in studies of acute lung injury (73). This inflammatory response leads to an increase in the expression levels of vascular endothelial adhesion molecules (such as VCAM 1, ICAM 1 and E-selectin), causing the inner walls of pulmonary microvessels to become highly adhesive. This creates conditions for the colonization of tumor cells, a phenomenon observed in colorectal cancer and COVID-19 (74, 75). Furthermore, the increase in branched-chain amino acid levels in the blood circulation, which modulates the immune microenvironment of various cancers, will further enhance the adhesiveness of the inner walls of blood vessels (76).

At the same time, the reprogramming of tumor cells leads to a decline in the immune surveillance ability of the pulmonary vascular microenvironment. Microbial products from the mucosal site of the primary tumor are transported through the bloodstream, driving neutrophils to accumulate in the lungs. As demonstrated in studies of fungal dysbiosis and allergic airway disease, disruption of the gut microbiota activates CX3CR1^+^ mononuclear phagocytes, further accelerating the enrichment of neutrophils (77). In models of lung metastasis, these neutrophils that are recruited to the lungs usually form neutrophil extracellular trapping nets. This structure not only functions like a net to capture tumor cells, but also blocks the killing actions of NK cells and CD8^+^ T cells, establishing a local immune barrier zone (78).

Meanwhile, the adaptive immune surveillance function is also disrupted. Extracellular signals from neutrophils and myeloid cytokines can promote the expansion of regulatory B cells (Bregs), and the IL-10 secreted by B cells can effectively inhibit the immune response of effector T cells (79). Meanwhile, the systemic signal disorder caused by dysbiosis damages the immune cells (such as mucosal-associated invariant T (MAIT) cells and γδT cells) that play a crucial monitoring role in the lung mucosa, and disrupts the balance between pro-inflammatory Th17 cells and Tregs that suppress immunity (80, 81). These changes collectively weakened the anti-tumor immune capacity of the lungs, creating a local microenvironment conducive to tumor metastasis.

### 3.2 The gut-liver axis: hepatic immune tolerance and fibrotic remodeling

As the main immune and metabolic filtering organ for portal vein return, the liver’s immune surveillance ability is weakened before metastatic tumor cells arrive, as established in the immunological landscape of primary and metastatic liver cancer (82). Through the intestinal-liver axis pathway, which serves as a critical mechanism in liver cancer pathogenesis, the signals generated by mucosal immune dysregulation are transmitted via the portal vein circulation, thereby systematically reprogramming the liver’s immunity before the occurrence of metastasis (83) (**Figure 2C**).

In colon cancer, the disruption of the intestinal vascular barrier leads to the continuous entry of intestinal bacteria, microbial metabolites, and tumor-derived exosomes into the portal vein, thereby triggering and maintaining a chronic portal vein inflammation state (63). Under the continuous and chronic inflammatory stimulation, the inherent Kupffer cells in the liver enter an endotoxin tolerance state, changing from the mode responsible for antibacterial surveillance to an immunosuppressive M2-like phenotype characterized by high expression of transforming growth factor-β and interleukin-10 (84, 85). This change actively reduces the immune surveillance function of the liver, transforming it from an organ that filters out abnormal cells to a place where CTCs can survive and remain for an early stage. The metabolic signals produced by intestinal flora imbalance further reinforce this state of immune tolerance. Specifically, secondary bile acids such as deoxycholic acid (DCA) and lithocholic acid (LCA), generated by specific bacterial metabolism, accumulate in the portal vein circulation. These metabolites activate TGR5 receptors primarily on Kupffer cells (55, 86). This activation promotes the polarization of Kupffer cells toward an immunosuppressive state and triggers the secretion of chemokines that actively recruit myeloid-derived suppressor cells (MDSCs) into the liver. These recruited MDSCs subsequently weaken the function of cytotoxic T cells, thereby amplifying the systemic immunosuppressive effect. In summary, these immune changes work together to gradually weaken the liver’s immune surveillance ability. This process is driven by signals released by both microorganisms and tumor cells, and it is a key adaptive mechanism before tumor metastasis to the liver.

Meanwhile, intestinal inflammation and metabolic disorders can also cause structural changes in the extracellular matrix of liver cells, making the liver more prone to the formation of metastatic foci. Disruption of bile acid homeostasis and chronic low-grade inflammation will activate hepatic stellate cells (HSCs), leading to early fibrotic changes and deposition of highly cross-linked collagen matrix as seen in liver fibrosis (87). This fibrotic remodeling significantly increases the hardness of liver tissue. The resulting biomechanical signals, through integrin-dependent mechanical conduction mechanisms, promote the colonization of DTCs by remodeling the hepatic biomechanical microenvironment (88). At the same time, it enhanced the adhesion, survival and proliferation capabilities of tumor cells upon their arrival (89). This biomechanical suitability and immunosuppressive liver microenvironmental coordination system function to promote tumor metastasis. For instance, pericytes and other stromal components form multicellular clusters with CTCs and neutrophils, thereby further facilitating endothelial cell migration and early metastasis and spread (90). Through the synergistic effect of the aforementioned immune-structural adaptation mechanisms, the intestinal-liver axis, as a component of the CMIS, simultaneously weakened the immune defense of the liver and reshaped its mechanical microenvironment, thereby creating an ideal colonization site for invading tumor cells (91).

### 3.3 The gut–brain axis: pre-emptive erosion of neuro-immune surveillance and barrier integrity

Studies have shown that although the brain has a protective blood-brain barrier (BBB), the innate immune state is continuously regulated by peripheral signals, including those from the intestinal mucosal immune system (92). Increasing evidence indicates that dysbiosis of the gut microbiota is associated with an increased incidence of brain metastases, especially in lung cancer and breast cancer (93–95). The susceptibility of the central nervous system to metastasis is not only influenced by local factors, but also regulated by distant mucosal immunity. These observations collectively reveal the existence of the gut-brain-metastasis axis: before CTCs reach the brain, the neuro-immune homeostasis has gradually been disrupted (92).

The main target of this distant immune regulation is the inherent macrophage population in the brain, namely microglia (**Figure 1D**). The dysbiosis associated with intestinal barrier dysfunction, often observed in inflammatory bowel disease and systemic tumor progression, promotes the spread of inflammatory mediators and microbial metabolites (including cytokines and tryptophan-derived products) throughout the body (92, 96). These cyclic signals may enter the central nervous system through the functionally impaired BBB, driving microglia to undergo reprogramming from an immune surveillance state to a phenotype that promotes tissue remodeling and tumor support, a process demonstrated in breast and lung cancer brain metastasis (97) (**Figure 2D**). Activated microglial cells will secrete matrix metalloproteinases (MMPs) and vascular endothelial growth factor (VEGF). These substances gradually weaken the tight junctions of the BBB and the integrity of the endothelium, as identified in cases of high-grade glioma and hypoxic microenvironments. This reduces the difficulty for subsequent tumor cells to penetrate the BBB and invade the brain parenchyma tissue (98–100). Meanwhile, the tryptophan metabolic pathway activated by the gut microbiota further drives microglia and local immune responses towards a tolerogenic state, rather than exerting cytotoxic defense functions, according to recent findings in brain cancer (101).

Disorder of the intestinal microbiota will further weaken the immune surveillance function of the nervous system. As documented in colorectal cancer research, changes in the metabolism of SCFAs and tryptophan disrupt the function of astrocytes, weakening their ability to maintain the integrity of the BBB and coordinate local immune defense (102–104). The reprogrammed astrocytes, in combination with activated microglia, establish an immunosuppressive neural microenvironment. This environment is characterized by reduced T cell infiltration, weakened effector functions, and increased secretion of inhibitory cytokines such as IL-10 (92). These changes collectively suppressed the immune surveillance function of the central nervous system, allowing tumor cells reaching the brain to evade the immune system and survive in a latent state. Furthermore, recent research in colorectal cancer and glioma has found that the gut microbiota can affect the efficacy of brain-targeted therapy, further demonstrating that this long-distance immune regulation has a significant impact on the tumor metastasis process (104, 105). Therefore, the occurrence of brain metastasis should not be simply regarded as the result of tumor cells suddenly breaking through the BBB. Rather, it should be considered as the manifestation of the long-term and gradual weakening of the original immune surveillance function of the nervous system. This weakening stems from chronic mucosal immune disorders and continues to exert an impact through the entire mucosal immune network throughout the body.

## 4 Dormancy and awakening: immune arbitration at mucosal metastatic niches

**Figure 3:**
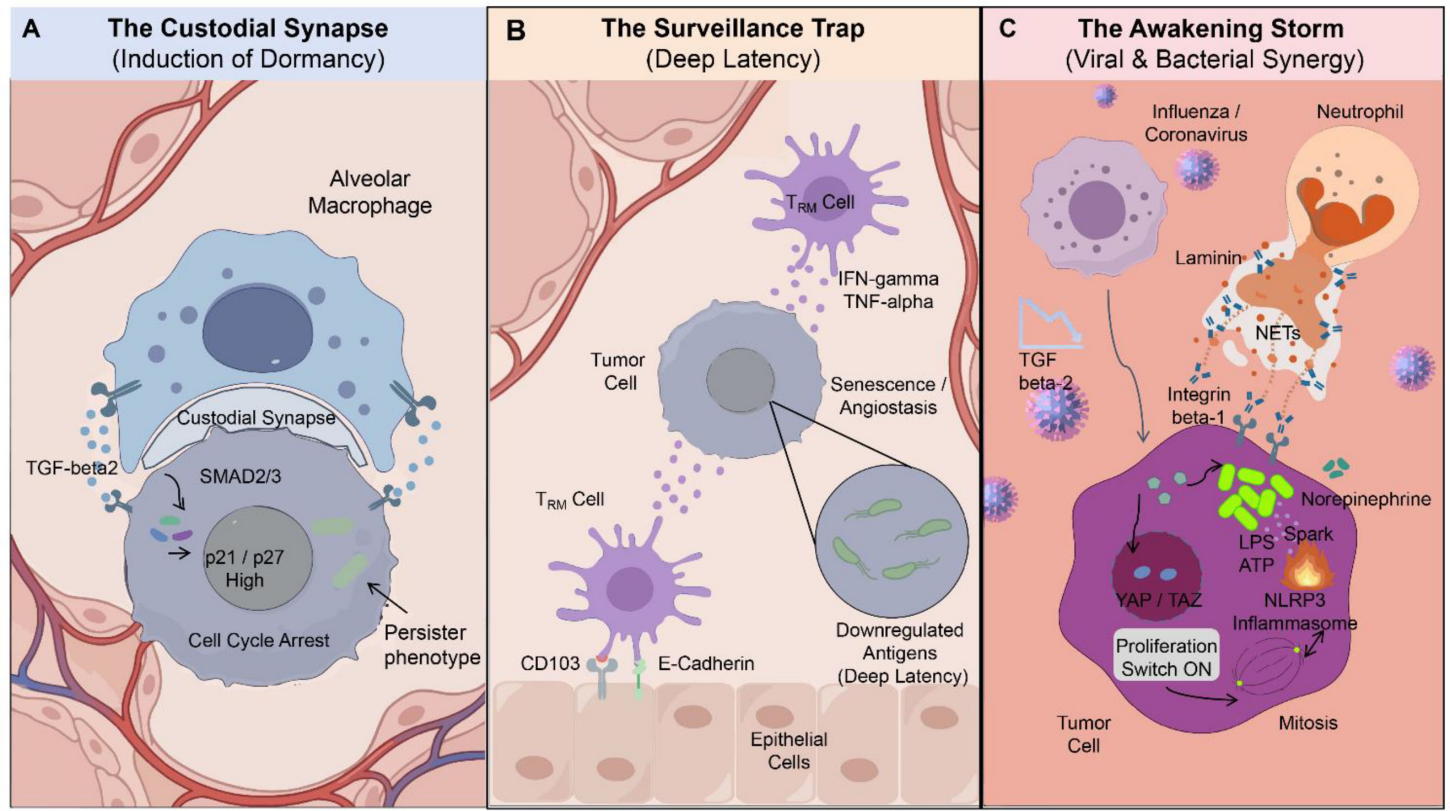
Mucosal immune control of metastatic dormancy and awakening within the microbe–tumor symbiont. **(A)** In the lung, alveolar macrophages (AMs) establish a sustained physical and signaling interface (“custodial synapse”) with disseminated tumor cells (DTCs). AM-derived TGF-β2 activates SMAD2/3 signaling in DTCs, inducing p21/p27 expression and stable cell-cycle arrest. Intracellular bacteria persist in a metabolically quiescent, low-immunogenic persister state, remaining compatible with immune-enforced dormancy. **(B)** Tissue-resident memory T (T_RM_) cells, anchored within the epithelium via CD103–E-cadherin interactions, provide continuous local surveillance. Tonic secretion of IFN-γ and TNF-α reinforces tumor cell senescence and suppresses angiogenesis. Concurrent bacterial antigen silencing within the symbiont maintains immune invisibility, stabilizing a state of deep metastatic latency. **(C)** Acute viral infection reprograms the metastatic niche, withdrawing AM-derived dormancy signals and inducing neutrophil recruitment and NET formation. NET-associated laminin engages integrin β1 on DTCs, activating YAP/TAZ-driven proliferative programs. In parallel, stress and inflammatory cues resuscitate intracellular bacteria, triggering metabolic activation, release of bacterial products (e.g., LPS, ATP), and inflammasome signaling. The convergence of stromal, immune, and microbial activation collapses dormancy and drives metastatic outgrowth.

Studies have shown that when microbial-tumor symbionts reach distant mucosal organs such as the lungs or liver, whether the tumor cells can survive and eventually form metastatic foci mainly depends on their interaction with the local mucosal immune microenvironment. Increasing evidence indicates that this interaction is not only regulated by the characteristics of the tumor cells themselves, but more fundamentally depends on the dynamic immune balance established by the resident innate and adaptive immune cells in the tissue (106–108). This balance system regulates three possible outcomes: the tumor cells are cleared by the immune system, enter a long-term dormant state, or gain proliferative advantages and activate the metastasis program. The presence of intracellular symbiotic microorganisms generates a persistent low-level immune disturbance, making the immune control unstable. Therefore, metastatic progression is not simply an extension of autonomous cell proliferation, but rather more like a continuous, unpredictable negotiation and confrontation between the mucosal immune system and the microbial-tumor symbiotic entity (**Figure 3**).

### 4.1 Contact-dependent dormancy enforcement by alveolar macrophages

As the organ where metastatic cells most commonly colonize, the lungs possess a unique innate immune system. For tumor cells that reach the lungs, alveolar macrophages (AMs) are the first line of defense they encounter. Besides being able to eliminate pathogens, evidence from liver and lung metastasis and primary lung cancer suggests that AMs can also maintain a local immunosuppressive state, thereby causing the metastatic tumor cells to enter a dormant state (109–111). Notably, recent *in vivo* imaging in breast cancer models revealed that AMs play a decisive regulatory role in the transition of metastatic tumor cells from a dormant state to a proliferative state (112) (**Figure 3A**).

AMs interact with DTCs through the regulation of synapses and transmit signals that induce dormancy to the tumor cells. The TGF-β2 protein secreted by macrophages activates the SMAD2/3-p38 MAPK signaling pathway within the tumor cells. This activation leads to an increase in the levels of cell cycle inhibitory proteins p21 and p27, and activates gene programs related to cell dormancy such as NR2F1 and SOX9 (113–115). Through this mechanism, macrophages create a stable quiescent state on the mucosal surface, mimicking the physiological process of immune tolerance. This effectively inhibits the proliferation of tumor cells, but does not trigger significant cell killing.

However, the existence of the microbial-tumor symbiosis introduces instability factors that hinder the maintenance of the dormancy mechanism. The bacterial components in it will generate continuous immune stimulation. For instance, microbial molecules such as lipopolysaccharide can continuously activate the pattern recognition receptors on the surface of macrophages (such as TLR4), forming a long-term, low-intensity chronic stimulation (116). This stimulation, although not strong enough to trigger acute inflammation, can gradually alter the functional state of macrophages, transforming them into pro-inflammatory types, thereby breaking the dormant state (117, 118).

This stimulation makes the pulmonary microenvironment extremely sensitive to external environmental disturbances. As demonstrated in studies of ovarian cancer and surgical models of breast and lung cancer, acute inflammatory events, such as respiratory tract infections, surgical stress, or tissue damage caused by treatment, can amplify the inflammatory signals from both microorganisms and the host, thereby disrupting the balanced state that maintains dormancy and restarting the cell cycle of DTCs (79, 119). Experimental evidence from lung adenocarcinoma models indicates that sterile inflammation can reactivate dormant tumor cells, and activating specific innate immune pathways (such as the STING signaling pathway) can reverse this activation process (120). Therefore, although macrophages serve as an important immune checkpoint in preventing tumor metastasis, the presence of intracellular symbiotic microorganisms may cause continuous chronic stimulation, creating conditions for tumor cells to evade immune surveillance and regrow in the future.

### 4.2 Antigen-threshold surveillance by tissue-resident memory T cells

Although macrophages are the first line of immune defense in maintaining tumor dormancy, to achieve long-term control of DTCs, ultimately, it is necessary to rely on immune surveillance that can recognize specific antigens. T cells that are tissue-resident memory cells are like “sentinels” permanently stationed within the tissues, constantly monitoring the changes in antigens in the mucosal tissues (121). The main mode of action of these T cells is not direct killing, but rather like “sensors”, monitoring the antigen levels, thereby maintaining immune balance. As long as the antigen levels do not exceed a critical point, they can continuously inhibit the growth of metastatic tumors (122) (**Figure 3B**).

The tissue-resident memory T (T_RM_) cells of the organism specifically express markers such as CD69 and CD103, and remain permanently in non-lymphoid tissues like the lungs and liver, as well as in mucosal barriers. They possess unique gene expression and functional characteristics (121). Immunocytes with high expression of CD103 can bind to E-cadherin on the surface of epithelial cells and the matrix, as identified in skin and brain models, thereby firmly anchoring themselves in the local microenvironment of the tissue and achieving local immune surveillance (123–126). In the dormant micro-metastasis environment of melanoma and respiratory tissues, T_RM_ cells maintain the basal immune stress state by continuously secreting low levels of interferon-γ (IFN-γ) and tumor necrosis factor-α (TNF-α) (127–129). At the same time, it can inhibit the growth of tumor cells, prevent the formation of new blood vessels, and stabilize the surrounding matrix structure.

However, the bacteria that are colonized within tumor cells inhibit antigen presentation and inflammatory signal transduction by down-regulating the expression of highly immunogenic molecules such as flagellin and peptidoglycan (24, 130), maintain the antigen signal below the activation threshold of T_RM_ cells (131, 132). This dormant state of the bacteria enables the symbiotic bacteria to evade the immune surveillance of T_RM_ cells, thereby avoiding subsequent immune killing responses (133). More importantly, the bacteria within tumor cells can sense the dynamic changes in the local mucosal environment through their inherent biological sensing mechanisms (such as changes in nutrient supply, inflammatory intensity, or host stress signals), maintaining this balance (134, 135).

The combined action of T_RM_-mediated immune surveillance and the antigen silencing of microorganisms establishes a dynamic “escape balance” in the mucosal microenvironment. On one hand, T_RM_ maintain tumor cell dormancy through local non-cell-dissolving isolation; on the other hand, symbiotic microorganisms actively regulate their metabolism and antigen expression to maintain immune concealment. However, this balance is inherently very fragile. Local mucosal infections or systemic dysbiosis can trigger inflammation, which may have a dual effect: on the one hand, it weakens the surveillance function of T_RM_; on the other hand, it activates the symbiotic microorganisms that were previously in a resting state, thereby disrupting the balance and leading to immune escape and tumor recurrence.

### 4.3 Breaching the immune threshold: viral infection as a systemic trigger of dormancy collapse and metastatic awakening

The latest study by Chia SB et al. (136) indicates that acute respiratory viral infections, as a powerful systemic trigger factor, can simultaneously disrupt the dormant mucosal immune program of metastatic breast cancer (136). The starting point of immune response disruption by pathogens such as influenza or SARS-CoV-2 is the host’s immune reaction to these viral pathogens. During acute infection, the alveolar macrophages that maintain the dormancy of tumor cells, as documented in breast cancer and head and neck squamous cell carcinoma, will transform into an anti-viral state with high expression of type I interferons and pro-inflammatory factors, and will be unable to continue to maintain the dormancy of tumor cells (112, 113, 115). Furthermore, the acute systemic physiological stress induced by viral infection triggers the massive release of norepinephrine, a critical neuro-endocrine mediator illustrated in **Figure 3C**. Norepinephrine binds to β2-adrenergic receptors on both neutrophils and dormant tumor cells, which has been shown to stimulate the release of S100A8/A9 and significantly facilitate the recruitment of neutrophils (112, 129).Meanwhile, viral infection leads to the recruitment of a large number of neutrophils, thereby forming neutrophil extracellular traps (NETs). The matrix components related to NETs (especially laminin) bind to the integrin receptors on the surface of dormant tumor cells. Activate the YAP/TAZ transcription switch and other signaling pathways, restart the tumor cell cycle and restore its proliferative function, a mechanism identified in hepatocellular carcinoma and various mechanotransduction models (137–139) (**Figure 3C**).

For the microbial-tumor symbiosis, its dual response to the host inflammatory signals further exacerbates this immune disorder. The microenvironment characterized by elevated levels of type I interferons and IL-6 during acute viral infection can awaken dormant, metabolically inactive intracellular bacteria, causing them to re-express pathogen-associated molecular patterns with immunogenicity and release pro-inflammatory factors, thereby enhancing local innate immune activation and matrix remodeling, accelerated the recurrence process of the tumor (27, 47, 130).

The clinical treatment of viral respiratory infections may further exacerbate this process. When dealing with severe viral infections, the commonly used broad-spectrum antibiotics can cause significant systemic dysbiosis, disrupting the immune interaction regulation between the gut and the lungs (140). This imbalance will interfere with the functioning of memory T cells that reside in lung tissues, weaken the immune surveillance function mediated by these T_RM_ cells, and promote the resumption of microbial metabolism. As a result, it becomes a multiple catalyst for the collapse of immune balance (131).

A large-scale retrospective cohort analysis revealed that the rates of distant recurrence for breast cancer and lung cancer showed significant seasonal fluctuations, with their peak periods coinciding closely with the periods of influenza and coronavirus epidemics (136, 141). During the period of high community transmission of respiratory viruses and the subsequent stages, the number of newly diagnosed metastatic cases has increased, which is consistent with the hypothesis that infection may disrupt the dormant tumor microenvironment (142). Furthermore, cancer survivors who had suffered from severe respiratory infections (including influenza or COVID-19) had a significantly higher risk of developing advanced metastatic recurrence compared to the uninfected matched control group (143). The above research indicates that acute mucosal viral infections can transform the long-standing latent state of metastasis into an overt disease.

Notably, while current high-impact evidence primarily emphasizes respiratory viral infections awakening dormant cells in the lungs (136), this infection-driven awakening mechanism may represent a more general systemic phenomenon applicable to other mucosal-associated organs. For instance, in colorectal cancer (CRC), intestinal dysbiosis or localized mucosal infections can trigger inflammatory cascades through the gut-liver axis, leading to the recruitment of immunosuppressive cells and the potential reactivation of dormant DTCs in the liver (79, 110). Similarly, peritoneal inflammation has been linked to the recruitment of NET-forming neutrophils that reactivate dormant cells in the omental niche, as observed in ovarian cancer models (70). These observations, combined with recent reviews on viral-mediated metastasis (142), suggest that any severe mucosal infection can serve as a systemic “alarm” that disrupts the local immune-tolerance threshold across various metastatic niches, regardless of the primary cancer type.

Based on this theory, the period of high prevalence of respiratory viruses may be accompanied by an increased susceptibility to tumors. During this period, the dormant microbial-tumor symbiotic entities are most likely to be activated.

## 5 Translating mechanisms into strategies: a mechanism-guided mucosal intervention framework

Currently, the focus of tumor treatment lies in the proliferating tumor cells or the systemic immune system, while neglecting the mucosal immune environment and the interorgan interaction axes that play a dominant role in the initiation, dormancy and reactivation of metastasis (54). Previous studies have shown that the occurrence of metastasis is not due to a sudden failure of local defense in the body, but rather through continuous communication of the systemic mucosal immune network among different organs (14, 54). This network enables primary tumors to gradually and pre-emptively weaken the defense capabilities of the target organs by orchestrating the formation of the pre-metastatic niche (PMN) long before the arrival of disseminated tumor cells (62).

Therefore, effective anti-metastasis interventions should not be limited to traditional cytotoxic drugs or systemic immune stimulation. Instead, a strategy based on the overall regulation of the mucosal system is required. To clarify the translational landscape of these interventions, we categorize them into two distinct developmental stages: emerging preclinical innovations that target specific mechanistic vulnerabilities (**Table 1**) and clinically investigated strategies currently undergoing patient trials (**Table 2**). This framework encompasses three primary engineering approaches: Ecological, Immune, and Chemical-Biological Engineering.

**Table 1:** Emerging preclinical strategies targeting mucosal immunity in metastasis.

Engineering strategy	Intervention/modality	Target mechanism	Preclinical rationale (murine models)
Ecological Engineering	Engineered Probiotics (e.g., E. coli Nissle 1917)	Gut-Lung/Gut-Liver Axis	Releasing immunomodulatory metabolites (e.g., SCFAs) or checkpoint inhibitors directly in the gut to remotely enforce barrier integrity and suppress pre-metastatic niche formation.
Ecological Engineering	Dietary Tryptophan Modulation	AhR Signaling Pathway	Enhancing AhR ligand availability to maintain the function of ILC3s and repair epithelial barriers, preventing microbial translocation and systemic inflammation.
Chemical-Bio Engineering	Glycan-Editing Enzymes (e.g., Sialidase-conjugates)	Hypersialylation/Siglec Axis	Targeted “desialylation” of tumor surfaces strips the “don’t eat me” sialic acid signals, reactivating NK cell-mediated cytotoxicity and preventing immune evasion.
Chemical-Bio Engineering	Bacteria-Functionalized Nanoparticles	Intratumoral Microbiota	Utilizing facultative anaerobic bacteria to penetrate hypoxic tumor cores and deliver chemotherapeutic payloads or STING agonists that are otherwise inaccessible to systemic drugs.
Immune Engineering	Mucosal Nanovaccines (Nasal/Oral)	Tissue-Resident Memory T Cells (T_RM_)	Inducing long-lasting T_RM_​populations specifically in the lungs or gut mucosa. These “sentinel” cells provide immediate protection against metastatic colonization upon antigen re-exposure.
Immune Engineering	Bio-Hybrid Delivery Systems (e.g., Neutrophil-Hitchhiking)	Pre-metastatic Niche Inflammation	Leveraging the natural homing ability of neutrophils to inflammatory sites to transport therapeutics across physiological barriers (e.g., Blood-Brain Barrier) to target brain metastasis.

**Table 2:** Clinically investigated interventions and active trials.

Engineering strategy	Target/intervention	Trial ID	Phase	Key rationale
Immune Engineering	Inhaled mRNA Vaccine	NCT06928922	Early Phase 1	Directly establishing mucosal immunity (T_RM_/IgA) in the lung niche to intercept metastasis.
Ecological Engineering	FMT + Anti-PD-L1	NCT05502913	Phase 2	Investigates remodeling the gut microbiome to overcome resistance to immunotherapy in lung cancer, validating the gut-lung axis.
Ecological Engineering	FMT + Atezolizumab/Bev	NCT05690048	Phase 2	Specifically targets the gut-liver axis to reverse immune tolerance (endotoxin tolerance) in liver macrophages (Kupffer cells).
Ecological Engineering	FMT from Responders	NCT03356860	Phase 1 Phase 2	A landmark study demonstrating that gut microbiota composition determines the efficacy of systemic immune checkpoint blockade.
Ecological Engineering	Oral FMT Capsules	NCT04729322	Phase 2	Uses Oral Capsules for non-invasive, repeated delivery to maintain barrier repair in metastatic colorectal cancer.
Chemical-Bio Engineering	Intratumoral *C. novyi-NT*	NCT03435952	Phase 1	Uses anaerobic bacterial spores to colonize hypoxic tumor cores and induce bacteriolysis
Chemical-Bio Engineering	Intratumoral SYNB1891	NCT04167137	Phase 1	Engineered *E. coli* acting as a “Trojan Horse” to deliver STING agonists directly into the tumor niche.
Chemical-Bio Engineering	Intratumoral Oncolytic Virus	NCT05868707	Phase 3	Uses OH2 virus to selectively replicate in and lyse tumor cells, releasing antigens and disrupting the physical tumor structure.

### 5.1 Mapping actionable vulnerabilities along the mucosal immune axis

During the multi-stage process of tumor metastasis, the symbiotic relationship formed between microorganisms and tumor cells collaboratively promotes the formation of the pre-metastatic microenvironment, the systemic dissemination of tumors, and the reactivation of dormant cells through systemic axes such as the gut-lung, gut-liver, and gut-brain axes (62, 97, 136). This process is not only related to the tumor cells themselves, but more importantly, the mucosal immune surveillance function between different organs throughout the body is gradually being weakened and altered, a phenomenon documented in colorectal cancer liver metastasis and broader metastatic frameworks (14, 54, 55). This dysfunction throughout the mucosal immune system reveals multiple vulnerable points at the tissue level, mainly including: damage to the protective barrier caused by disrupted intestinal flora, reduction or dysfunction of the resident “immune sentry” cells in the tissues, the persistent presence and active role of symbiotic microorganisms within tumor cells, and the unexpected “activation” of dormant tumor cells by inflammatory signals, as highlighted in the context of lung cancer and engineered biological systems (144, 145). Based on the above mechanism, we have integrated the therapeutic framework of the mucosal immune axis (see **Table 3**), which matches specific pathological functions with corresponding intervention points, aiming to stabilize the immune balance and block the progression of metastasis.

**Table 3:** Therapeutic framework of the mucosal immune axis.

Therapeutic target	Proposed intervention strategy
Dysbiosis and systemic metabolite deficiency	Precision probiotics, prebiotics, or fecal microbiota transplantation to restore eubiosis (66, 167, 168) Administration of SCFA prodrugs or AhR ligands to re−establish immune tolerance (69)
Pathogenic virulence factor production	Monoclonal antibodies or small−molecule inhibitors targeting specific bacterial virulence factors (60, 169).
NETs formation	Degradation of NETs with DNase I or inhibition of their formation with PAD4 inhibitors; a potent combinatorial approach with immunotherapy (170)
Establishment of the immunosuppressive PMN	Combination of ICIs with agents that target immunosuppressive myeloid cells or restore innate lymphocyte function (65, 170)
Dysregulated gut-lung axis signaling	Intrapulmonary or oral delivery of vaccines/immunomodulators leveraging mucosal homing for site−specific immunity (168, 171); Polysaccharides from medicinal herbs to rebalance the gut−lung ecosystem, reduce inflammation, and restore barrier integrity (81, 172, 173).
Dysbiosis and pathogenic bacterial translocation	Probiotics (e.g., Lactobacillus spp.) to enhance barrier function and correct dysbiosis; adjunctive use of the gut−selective antibiotic rifaximin to reduce bacterial overgrowth/translocation with minimal systemic exposure (174, 175); Global reset of a dysbiotic ecosystem to ameliorate downstream hepatic inflammation (176–178).
Reprogramming of hepatic immune cells	Combination of ICIs with agents that repolarize Kupffer cells or restore dendritic cell function (179); Blockade of IL-10 or TGF-β signaling to reverse immunosuppression; Administration of microbial metabolites (e.g., AhR ligands) to support innate lymphocyte function (180).
Systemic metabolic and bile acid dysregulation	Ursodeoxycholic acid or farnesoid X receptor agonists to restore physiologic bile acid homeostasis (181); Supplementation with protective microbial metabolites (182); Targeting activated HSCs to prevent extracellular matrix stiffening (178).
Intestinal barrier and mitochondrial dysfunction	Nutrients or agents that enhance enterocyte mitochondrial function (178); Compounds such as green tea polyphenols or specific amino acids demonstrated to improve intestinal mucosal immunity and barrier integrity (183, 184)

### 5.2 Engineering mucosal equilibrium to suppress metastasis

Based on this framework, effective anti-metastasis intervention needs to be implemented before the tumor takes the dominant position. The key lies in restoring the regulatory function of mucosal immunity and the balance of the ecosystem, rather than merely targeting the proliferating tumor cells. To clarify the translational landscape, we categorize these interventions into emerging preclinical innovations (**Table 1**) and clinically investigated strategies (**Table 2**).

In preclinical settings (**Table 1**), the primary goal is to address functional disorders caused by intestinal flora imbalance. The currently developed engineered probiotics and living biological therapeutic preparations can enhance the strength of the intestinal epithelial barrier, regulate the mucosal immune state, and block the signals that promote tumor metastasis through the intestinal-lung and intestinal-liver pathways (144–147). These designed biological treatment systems can release immune-regulating substances (such as immune checkpoint inhibitors or anti-inflammatory factors) at the local tumor or mucosal areas, thereby intercepting the signals that promote metastasis at the source and minimizing the toxic effects on other parts of the body as much as possible (148–150). Meanwhile, the nano-treatment platform and the biological hybrid delivery system can further enhance the penetration ability of the drug through the mucus layer and fibrotic barrier, thereby improving the delivery efficiency to the mucosal and lymphatic microenvironment (151, 152). Transitioning to clinical validation (**Table 2**), current efforts focus on ecosystem restoration via Fecal Microbiota Transplantation (FMT). Clinical trials such as NCT05502913 (Lung Cancer) and NCT05690048 (Hepatocellular Carcinoma) are actively investigating whether remodeling the gut microbiome can reverse immune tolerance and overcome resistance to immunotherapy, validating the gut-target organ axes in humans.

Secondly, the focus of the immune enhancement strategy is to restore the functions of the resident immune cells in the tissues that are responsible for controlling tumor dormancy and maintaining immune balance. Preclinically (**Table 1**), vaccination administered through mucosal routes such as nasal or oral administration has unique advantages: it can induce long-term resident immune cells and secretory antibodies in the local organs prone to metastasis, thereby forming local immune memory (153, 154). These “permanent sentinels” can quickly identify and respond to the revival signals of tumors and inflammatory disturbances. When tiny metastatic foci are infected or the body is awakened by stress, they provide timely and effective protection (155). With the continuous development of nanoparticle vaccine delivery technologies and new mucosal adjuvants, the clinical application prospects of this strategy are becoming increasingly broad (156–158). In the clinical realm (**Table 2**), this concept is advancing through trials like the inhaled mRNA vaccine (NCT06928922), which aims to establish sentinel immunity directly in the pulmonary niche to intercept metastatic recurrence.

The third type of strategy is selective biological intervention, where the target is not the tumor cells themselves, but rather the “supporting structures” that facilitate tumor metastasis. Preclinical approaches (**Table 1**) utilize precise antibacterial strategies to eliminate tumor-associated bacteria without the widespread disruption caused by broad-spectrum antibiotics (159–161). Meanwhile, therapeutic approaches aimed at removing specific sugar chains are expected to help the immune system re-identify tumor cells and prevent CTCs from adhering to the walls of blood vessels by interfering with the interactions between PD-L1, CD24 or mucin-selectin (162–165). The latest research indicates that the immune checkpoint molecules that have undergone desialylation treatment may have a better effect in predicting the efficacy of immunotherapy (164, 166). Moving to clinical application (**Table 2**), strategies such as the intratumoral injection of C. novyi-NT (NCT03435952) and engineered E. coli (NCT04167137) have entered clinical trials. These therapies validate the concept of using bacteria not just as targets, but as active agents to disrupt the tumor-microbe symbiosis and reignite anti-tumor immunity.

## 6 Current limitations and unresolved questions

While this review presents an ambitious and integrative model linking mucosal immunity to cancer metastasis, several limitations and unresolved questions remain in the field that warrant further exploration. Addressing these challenges will enhance the depth and applicability of mucosal immune-based interventions in cancer therapy. The following key areas require continued investigation:

### 6.1 Technical limitations in research

Despite growing evidence of the role of microbiota-tumor interactions, the precise molecular mechanisms underlying this phenomenon remain poorly understood. The complexity of microbial communities and the heterogeneity of tumor microenvironments pose challenges in defining the specific interactions that drive metastasis. More experimental data are needed to elucidate the precise mechanisms by which different microbial species influence tumor progression in various cancer types.

### 6.2 Detection challenges

Current methodologies, such as high-throughput sequencing, are limited in their ability to accurately detect and differentiate between tumor-associated microbes and resident microbiota. The sensitivity and specificity of these technologies need further improvement to enhance the accuracy of microbiome analyses in cancer research.

### 6.3 Clinical research limitations

While many mucosal immune intervention strategies have shown promise in preclinical studies, they remain largely unvalidated in clinical settings. Clinical trials are essential to evaluate the safety, efficacy, and long-term benefits of these strategies. However, many of these approaches are still in early stages of clinical testing, and substantial evidence is needed to confirm their clinical applicability.

### 6.4 Lack of clinical diversity

Most clinical trials investigating mucosal interventions are focused on a few cancer types, such as lung cancer and colorectal cancer. There is a need to expand clinical research to include other cancer types and assess the universality of mucosal immune interventions across different malignancies.

### 6.5 Challenges in biomarker development and personalized treatment

While several biomarkers reflecting mucosal immune destabilization have been proposed, their accuracy, specificity, and sensitivity are not yet fully established. Reliable biomarkers are critical for assessing mucosal immune status and tailoring interventions. Further validation is required to identify biomarkers that can predict treatment outcomes and guide personalized therapies.

### 6.6 Personalized mucosal immunotherapy

Given the diversity in individual microbiomes and immune responses, developing personalized mucosal immunotherapies remains a significant challenge. Tailoring interventions based on an individual’s unique microbiome profile and immune system will be crucial for improving treatment outcomes, but this requires more sophisticated diagnostic tools and models.

### 6.7 Barriers to translational research

Despite promising findings in preclinical models, translating mucosal immune interventions from the laboratory to clinical practice remains difficult. Several obstacles, including technological and methodological barriers, and ethical considerations, hinder the smooth transition of these interventions to human trials. Overcoming these challenges is essential for the successful integration of mucosal immunity strategies into cancer therapies.

In summary, while mucosal immunity represents a promising avenue for cancer therapy, significant hurdles remain. Future research must address these unresolved questions and limitations to fully realize the potential of mucosal immune interventions in clinical oncology.

## 7 Conclusion

A large number of studies have shown that the genetic instability, morphological plasticity and sterile inflammation of tumor cells are the fundamental causes for their spread. However, what ultimately determines whether these cells can survive, colonize and form metastasis is the key regulation of the mucosal immune system. This review systematically elaborates on the role and mechanism of mucosal immunity in various stages of tumor metastasis. During the process of tumor metastasis, various mechanisms such as microbial flora imbalance, disruption of the mucosal immune barrier, and imbalance of immune regulation interact with each other, leading to a continuous process where local imbalance gradually expands into a systemic immune remodeling throughout the body. In mucosal tissues, the interaction between the immune system and the microbiota plays a crucial role in determining the survival of tumor cells, regulating the permeability of the barrier, and transmitting immune signals to distant organs through an axis centered on the intestine, thereby enabling tumor colonization and metastasis.

At the primary lesion stage, the disruption of the mucosal barrier and the imbalance of immune function provide specific bacterial communities with the opportunity to enter tumor cells and establish long-term colonization. Over time, a stable microbial-tumor symbiosis is eventually formed. Furthermore, the glycosylation structure on the cell surface undergoes remodeling. Once the tumor cells enter the circulatory system, this characteristic enables the tumor cells to achieve immune evasion, continue to proliferate and spread. Meanwhile, this restructured immune signal will, through the common communication network of the mucosal system, send signals to distant organs through pathways such as the gut-lung axis, gut-liver axis, and gut-brain axis in advance, jointly weakening the barrier integrity and immune effect function of the distant organs, while reshaping the blood vessels and microenvironment of the tissues, making them exhibit strong inhibitory properties and sluggish inflammatory responses, thereby creating conditions for the subsequent retention and colonization of tumor cells.

In the distant organs where tumor cells have established colonies, the mucosal immune system plays a crucial regulatory role in the dormancy or proliferation of tumor cells. AMs and T_RM_ maintain the dormant state of tumor cells through specific molecular signals (such as TGF-β2 and CD103). The continuous low-intensity immune disturbances caused by intracellular symbiotic microorganisms keep this balance in an unstable state. When there is an acute mucosal immune disruption (such as respiratory virus infection), macrophages will transform into pro-inflammatory types, and the immune surveillance ability of T_RM_ will also weaken. The pro-inflammatory and matrix remodeling signals synergistically activate the proliferation pathway, thereby enabling the tumor cells to continue proliferating. This theory offers a possible explanation for the common clinical phenomenon of tumor recurrence after infection.

These findings underscore the necessity of constructing a new paradigm for cancer prevention, control, and treatment, one that is centered around mucosal immunity. Future strategies should not only focus on eliminating disseminated tumor cells (DTCs) but also work to stabilize mucosal immune surveillance, maintain epithelial barrier integrity, and prevent immune imbalance driven by chronic inflammation.

To aid in early detection and personalized treatment, it is crucial to develop diagnostic tools that can assess mucosal immune status. This may include the analysis of biomarkers such as SIgA profiles, which reflect the functional state of mucosal immune responses, and markers of tissue-resident immune cells like CD69 and CD103, which indicate immune activation or exhaustion within mucosal tissues. Additionally, microbial metabolites, such as short-chain fatty acids (SCFAs) and secondary bile acids, whose levels may reflect microbial dysbiosis and immune dysfunction at mucosal sites, could provide valuable insights into the immune landscape. These biomarkers, integrated into diagnostic panels, could assist in the early identification of minimal residual disease and potential immune escape, allowing for timely therapeutic interventions.

At the therapeutic level, strategies aimed at enhancing tissue-resident immunity, reshaping the mucosal immune microenvironment, and selectively disrupting immune escape mechanisms mediated by microorganisms and glycosylation processes may help maintain long-term tumor dormancy. Restoring the regulatory capacity of the mucosal immune system could reshape metastasis as a dynamic process that may be modulated by mucosal immune mechanisms. This approach offers new possibilities for improving cancer prognosis and enhancing the effectiveness of metastatic cancer therapies.

In conclusion, this review highlights the central role of mucosal immunity in cancer metastasis, emphasizing how mucosal immune dysregulation and microbial imbalances contribute to tumor progression and immune evasion. By restoring mucosal immune function and targeting microbial-tumor interactions, novel therapeutic strategies can be developed to manage metastasis more effectively. Additionally, the identification of specific biomarkers for mucosal immune status offers promising avenues for early detection and personalized treatment. As research advances, these insights may lead to transformative approaches in cancer treatment, turning metastasis from an inevitable event into a dynamic process that can be regulated and controlled.
